# Malformations of Core M3 on α-Dystroglycan Are the Leading Cause of Dystroglycanopathies

**DOI:** 10.1007/s12031-025-02320-z

**Published:** 2025-02-25

**Authors:** Wessam Sharaf-Eldin

**Affiliations:** Medical Molecular Genetics Department, National Research Centre, Cairo, Egypt

**Keywords:** Dystroglycanopathies, Neuromuscular diseases, O-Mannosylation, α-Dystroglycan, *POMGNT1*

## Abstract

Dystroglycanopathies (DGPs) are a group of autosomal recessive neuromuscular diseases with significant clinical and genetic heterogeneity. They originate due to defects in the O-mannosyl glycosylation of α-dystroglycan (α-DG), a prominent linker between the intracellular cytoskeleton and the extracellular matrix (ECM). Fundamentally, such interactions are crucial for the integrity of muscle fibers and neuromuscular synapses, where their defects are mainly associated with muscle and brain dysfunction. To date, biallelic variants in 18 genes have been associated with DGPs, where the underlying cause is still undefined in a significant proportion of patients. Glycosylation of α-DG generates three core motifs where the core M3 is responsible for interaction with the basement membrane. Consistently, all gene defects that corrupt core M3 maturation have been identified as causes of DGPs. *POMGNT1* which stimulates the generation of core M1 is also associated with DGPs, as it plays a central role in core M3 processing. Other genes involved in the glycosylation of α-DG seem unrelated to DPGs. The current review illustrates the *O*-mannosylation pathway of α-DG highlighting the functional properties of related genes and their contribution to the progression of DPGs. Different classes of DPGs are also elaborated characterizing the clinical features of each distinct type and phenotypes associated with each single gene. Finally, current therapeutic approaches with favorable outcomes are addressed. Potential achievements of preclinical and clinical studies would introduce effective curative therapies for this group of disorders in the near future.

## Background

Protein glycosylation is the covalent attachment of an oligosaccharide, also referred to as glycan, to the target protein by the action of glycosyltransferases. In mammals, these glycan chains are constituted of different combinations of ten distinct monosaccharides: glucose (Glc), N-acetylglucosamine (GlcNAc), glucuronic acid (GlcA), iduronic acid (IdoA), galactose (Gal), N-acetylgalactosamine (GalNAc), mannose (Man), xylose (Xyl), fucose (Fuc), and sialic acid (Sia) Brazil and Parkos 2022. Sia encompasses over 50 distinct species, including N-acetylneuraminic acid (Neu5Ac) and N-glycolylneuraminic acid (Neu5Gc) Zhu et al. 2024. Glycosylation represents one of the most prevalent and diverse posttranslational modifications (PTMs) crucial for protein structure and function. N-Glycosylation and O-glycosylation are the most common types of protein glycosylation He et al. 2024. In N-glycosylation, the sugar is connected to the nitrogen atom of an aspartate (Asp) residue; however, the carbohydrate moiety is attached to the oxygen atom of serine (Ser) or threonine (Thr) residues in O-glycosylation. Altered glycosylation patterns are associated with multiple human diseases, including congenital disorders of glycosylation (CDGs), diabetes, and cancer as well as inflammatory, autoimmune, and infectious diseases Reily et al. 2019.

Dystroglycanopathies (DGPs) are a distinct array of CDGs that occur due to defects in the O-mannosyl glycosylation of α-dystroglycan (α-DG). DG is encoded as a single polypeptide by the *DAG1* gene on chromosome 3p21 Kanagawa 2021. Subsequently, DG is cleaved into α- and β-DG subunits by autoproteolysis Akhavan et al. 2008. α-DG, a member of the dystrophin–glycoprotein complex (DGC), is a cell-surface receptor that binds ECM proteins including laminin-211, perlecan, and agrin in muscles, neurexin in the brain, and pikachurin in the eye (Ervasti and Campbell 1993, Ibraghimov-Beskrovnaya et al. 1992, Lu et al. 2020). The ability of α-DG to act as a receptor relies on its O-glycosylation, particularly at distinct Ser residues. According to the type of glycosidic linkage and structure of attached glycan, α-DG forms three different core motifs: core M1, core M2, and core M3. The core M3 is responsible for α-DG binding to the ECM proteins Jahncke and Wright 2023a.

α and β subunits of DG are non‐covalently linked to each other, where the C-terminus of α-DG interacts with the N-terminus of β-DG (10). β-DG is a transmembrane protein whose cytoplasmic C-terminus binds dystrophin, which in turn associates with the cytoskeleton Yoshida-Moriguchi and Campbell 2015. Based on their cellular localization, proteins of the DGC are divided into extracellular (α-DG), transmembrane (β-DG, sarcoglycans, sarcospan), and cytoplasmic (dystrophin, dystrobrevin, syntrophins, neuronal nitric oxide synthase). The DGC complex acts as a bridge between ECM and cytoskeleton in different tissues such as muscles, nervous system, lung, and kidney accounting for their proper morphology and normal physiology Gao and McNally 2015.

## Posttranslational Modifications of α-DG

α-DG consists of three domains: the α-DG N-terminal domain (α-DGN; 1–312 aa), a Ser/Thr-rich mucin-like domain (MLD; 313–485 aa), and a C-terminal domain (486–653 aa). α-DGN is normally cleaved at the sequence RVRR (309–312 aa) by the proprotein convertase furin without disturbing α-DG function. Before its separation, α-DGN facilitates O-glycosylation of MLD via interaction with the glycosyltransferase LARGE1 Yoshida-Moriguchi et al. 2010. α-DGN has also been found to have a protective role against influenza A virus (IAV) infection Greef et al. 2019. The N- and C-terminal domains contain several N-glycosylation sites; however, MLD contains at least 21 O-glycosylation sites Nilsson et al. 2010. α-DG is heavily glycosylated. Its amino acid sequence predicts a molecular weight of ~ 74 kDa; however, its apparent molecular weight (MW) varies from 100 to 200 kDa across tissues. The glycosylation status of α-DG is firmly controlled according to developmental phase and tissue type Endo 2014.

**Fig. 1:**
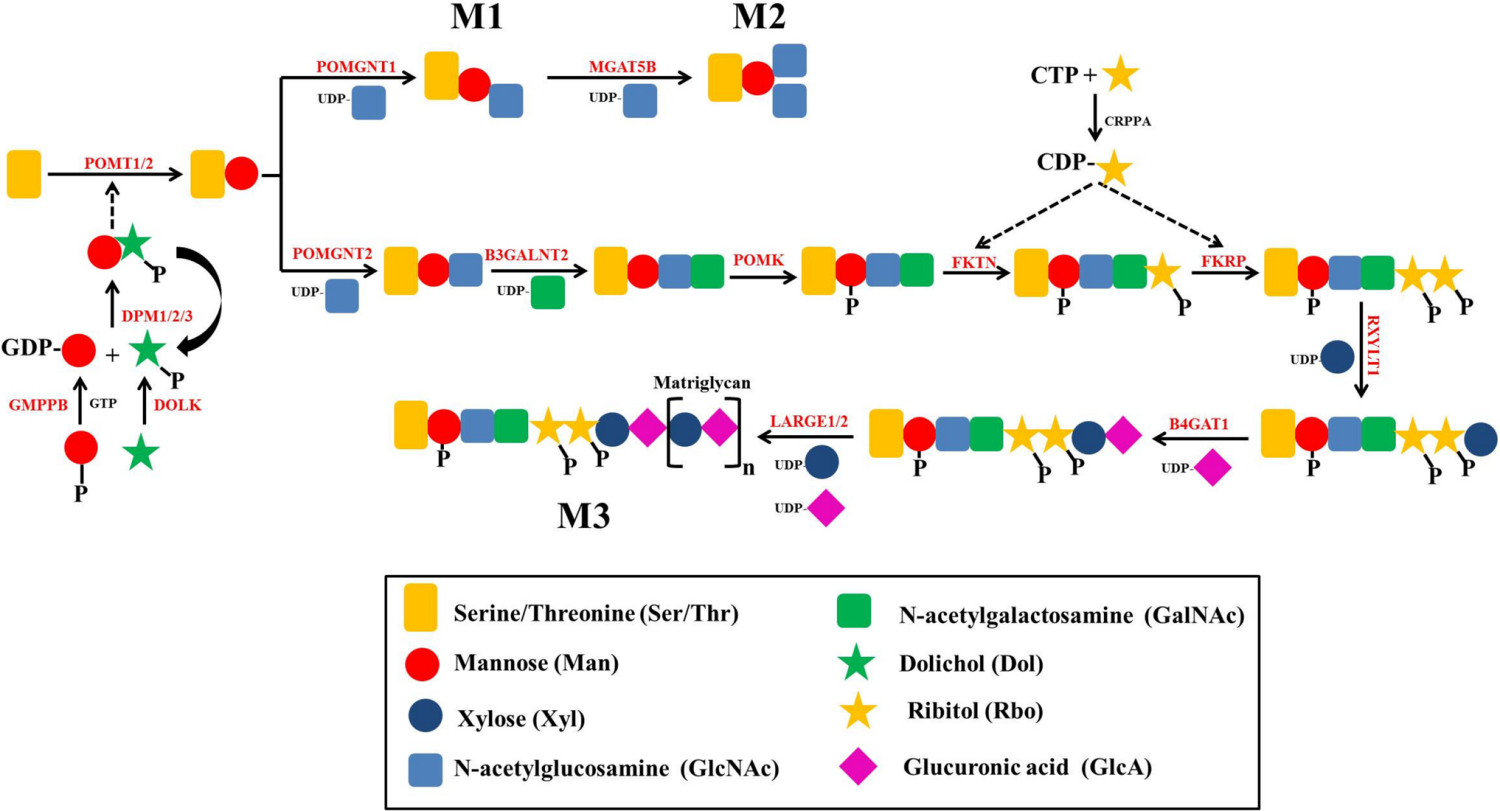
Synthesis of different core motifs on α-DG. Sugars are symbolled as circles. However, sugar derivatives are symbolled as squares (amino sugars), stars (alcohol sugars), and rhombus (sugar acids). Enzymes are indicated in red color

The O-glycosylation process of α-DG takes place in the endoplasmic reticulum (ER) and the Golgi apparatus (GA) requiring the harmonic activity of dozens of enzymes (Endo 2014) **(**Fig. 1**)**. It starts with the addition of O-Man by the ER-localized complex, protein O-mannosyltransferase (POMT) that catalyzes the transfer of a mannosyl residue (Man) to the hydroxyl groups of Ser or Thr residues of α-DG Bai et al. 2019. In humans, the POMT complex consists of two gene products, POMT1 and POMT2, where the co-expression of both genes is fundamental for the enzymatic activity Manya et al. 2004. Dolichol phosphate mannose (Dol-P-Man) serves as the major donor of mannosyl residues in the O-mannosylation of different proteins Tomita et al. 1998. Dol-P-Man is generated at the cytosolic face of the ER membrane by the dolichyl-phosphate mannosyltransferase (DPM) complex comprising three subunits: the cytoplasmic catalytic DPM1 and the transmembrane regulatory DPM2 and DPM3 Maeda and Kinoshita 2008. The DPM complex transfers Man from GDP-Man to the dolichol phosphate (Dol-P), where the synthesis of Dol-P and GDP-Man is catalyzed by dolichol kinase (DOLK) Kranz et al. 2007 and GDP-mannose pyrophosphorylase B (GMPPB) Chompoopong and Milone 2023, respectively.

**Fig. 2:**
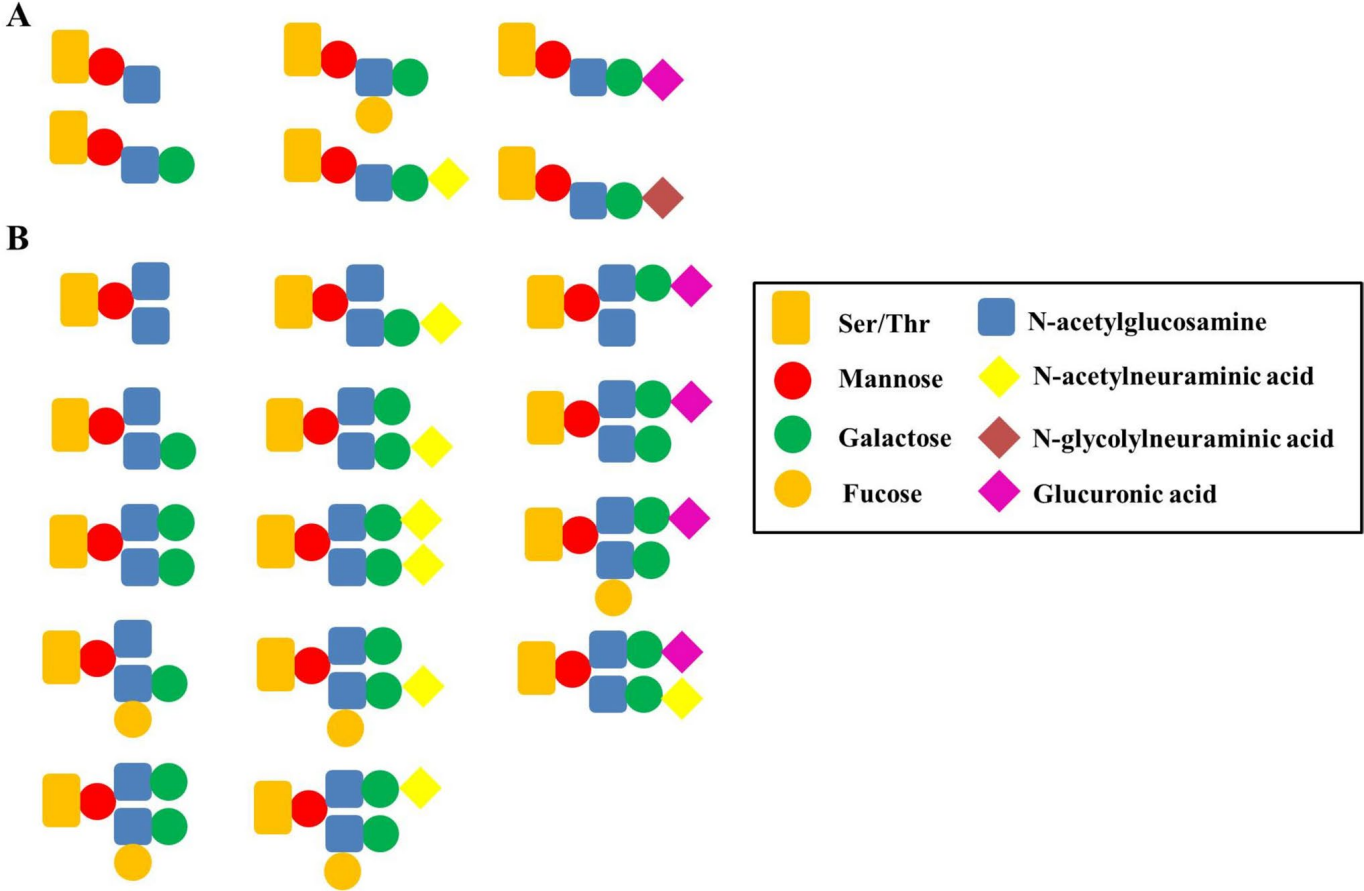
Different structures of core M1 (**A**) and core M2 (**B**). Sugars are symbolled as circles, amino sugars as squares, and sugar acids as rhombus

In the next step, N-acetylglucosamine (GLcNAc) is transferred from UDP-GlcNAc to the O-linked-Man generating three possible core motifs, (A) core M1, (B) core M2, and (C) core M3, based on the type of the formed glycosidic linkage. The core M1 is synthesized by protein O-linked-mannose β−1,2-N-acetylglucosaminyltransferase 1 (POMGNT1) that catalyzes the formation of β−1,2-glycosidic linkage between GLcNAc and Man Yoshida et al. 2001. The core M1 can be converted to core M2 by inserting another β−1,6-linked GlcNAc by the enzyme alpha-1,6-mannosylglycoprotein beta-1,6-N-acetyl-glucosaminyltransferase, isozyme B (MGAT5B) Inamori et al. 2004. In the GA, other monosaccharides can be added to cores M1 and M2 forming a variety of glycan structures using a series of glycosyltransferases, including galactosyltransferases, fucosyltransferases, glucuronyltransferases, and sialyltransferases Endo 2019. In mammals, at least six different structures of core M1 have been identified Praissman and Wells 2014, where the sialyl tetrasaccharide structure is the most abundant Meng et al. 2018**(**Fig. 2A**)**. In the context of core M2-based structures, at least 14 different mammalian structures have been found, where different branches can be added to the two GlcNAcs Praissman and Wells 2014**(**Fig. 2B**)**.

However, to generate the core M3, the β−1,4-glycosidic linkage is formed by the protein O-linked-mannose β−1,4-N-acetylglucosaminyltransferase 1 (POMGNT2) in the ER Manzini et al. 2012. Man-β−1,4-GLcNAc is a substrate for beta-1,3-N-acetylgalactosaminyltransferase 2 (B3GALNT2) that adds N-acetylgalactosamine (GalNAc) to the GlcNAc. Subsequently, protein O-mannose kinase (POMK) inserts a 6-linked phosphate (P) into the core Man. O-Man phosphorylation is crucial for subsequent ribitol-5-P (Rbo5P) insertion on core M3 by fukutin (FKTN) and fukutin-related protein (FKRP). Both FKTN and FKRP catalyze the same reaction (addition of Rbo5P), but to two different substrates, where FKTN transfers Rbo5P to the GalNAc, and FKRP adds another Rbo5P to the first Rbo. In both reactions, CDP-Rbo serves as the donor of RboP, where it is generated by CDP-L-ribitol pyrophosphorylase A (CRPPA) Kanagawa and Toda 2018. To the outermost Rbo, the glycosyltransferases ribitol xylosyltransferase 1 (RXYLT1) and β−1,4-glucuronyltransferase 1 (B4GAT1) add a xylose (Xyl) and a glucuronic acid (GlcA), respectively, using UDP-Xyl and UDP-GlcA as donor substrates. GlcA-Xyl disaccharide units, termed matriglycan, are then repeatedly inserted into the core M3 by the bifunctional glycosyltransferase LARGE1 or its paralog LARGE2 that exhibit dual xylosyltransferase and glucuronyltransferase activities Goddeeris et al. 2013. LARGE1 mediates glycosylation of α-DG in the brain, skeletal muscle, and heart, while LARGE2 mediates this process in the kidney, colon, and prostate Dietinger et al. 2020. Matriglycan serves as the binding site for ECM proteins containing laminin globular (LG) domains, including laminins, pikachurin, agrin, pikachurin, and the peplomer protein of distinct arenaviruses. Nearly, all α-DG binding proteins contain at least one LG domain, except for biglycan which can bind α-DG independent of its glycosylation Mamsa et al. 2022. The minimum length of matriglycan seems to be about four repeats, where longer chains enable α-DG to bind multiple proteins simultaneously Jahncke and Wright 2023b.

The core M1 and M2 glycans have been recognized on various mammalian proteins besides α-DG such as IgG2, phosphacan, CD24, neurofascin, and lecticans. However, the core M3 glycans seem specific for mammalian α-DG Endo 2019. The cores M1 and M2 do not mediate α-DG binding with ECM proteins (Combs and Ervasti 2005, Lee et al. 2012). However, the lack of core M1 structure was suggested to block core M3 processing, and the core M3 glycans are located in close proximity to core M1. Interestingly, POMGNT1 forms a complex with FKTN in the GA. First, POMGNT1 forms core M1 on an O-linked Man and then FKTN easily transfers Rbo5P to a neighboring core M3 structure (Kuwabara et al. 2016). Therefore, defects in POMGNT1 would corrupt the formation of POMGNT1–FKTN complex inhibiting the maturation of core M3 glycans. This could explain why the loss of core M1 glycans due to POMGNT1 deficiency correlates with DGPs, while variants in the genes encoding other enzymes involved in the synthesis of core M1 and M2 glycans have not been reported in patients with DGPs.

## Dystroglycanopathies (DGPs)

Dystroglycanopathies (DGPs) constitute a group of neuromuscular diseases with brain and eye anomalies. Symptoms range from congenital muscular dystrophy to adult-onset limb-girdle muscular dystrophy (LGMD). Serum levels of creatine kinase are markedly increased among patients. Muscle biopsies showed dystrophic features with hypoglycosylation of α-DG and defective laminin binding. Cardiomyopathy and respiratory distress are present in some cases. Using ultrasound imaging, affected patients might reveal multisystem anomalies in the prenatal setting Castro et al. 2024.

At least 18 genes have been correlated with DGPs. They are classified into three groups depending on the function of the affected gene: 1, primary DGPs due to variants in *DAG1* itself; 2, secondary DGPs due to variants in the genes that encode enzymes that catalyze the glycosylation of α-DG (*POMT1*, *POMT2*, *POMGNT1*, *POMGNT2*, *POMK*, *FKTN*, *FKRP*, *LARGE1*, *TMEM5*, *B3GALNT2*, or *B4GAT1*); 3, tertiary DGPs due to variants in the genes that encode enzymes involved in the synthesis and modification of donor substrates used by the α-DG modifying enzymes (*CRPPA*, *GMPPB*, *DPM1*, *DPM2*, *DPM3*, or DOLK) Song et al. 2021. Originally, variants in each DGP-related gene were thought to give rise to a clinically distinct disorder. However, it has been subsequently realized that variants in all DGP-related genes yield similar phenotypes that can be classified into three distinct groups according to their clinical severity, where the same gene can mediate different phenotypes **(**Table 1**)**. The determinant of phenotypic severity is to what extent the variant affects glycosylation of α-DG, where the phenotypic severity is inversely correlated with residual enzyme activity Mercuri et al. 2009.

**Table 1:** Genes associated with dystroglycanopathies and their correlated phenotypes according to the OMIM database

No	Gene (full name)—OMIM #	Disorder—OMIM #	Disorder previous nomenclatures	Gene previous symbols
Primary DGPs				
*1*	*DAG1* (dystroglycan 1)—128,239	MDDGA9—616,538		
		MDDGC9—613,818	LGMDR16, LGMD2P	
Secondary DGPs				
*2*	*POMT1* (protein O-mannosyltransferase 1)—607,423	MDDGA1—236,670		
		MDDGB1—613,155		
		MDDGC1—609,308	LGMDR11, LGMD2K	
*3*	*POMT2* (protein O-mannosyltransferase 2)—607,439	MDDGA2—613,150		
		MDDGB2—613,156		
		MDDGC2—613,158	LGMDR14, LGMD2N	
*4*	*POMGNT1* (protein O-linked-mannose β−1,2-N-acetylglucosaminyltransferase 1)—606,822	MDDGA3—253,280		
		MDDGB3—613,151		
		MDDGC3—613,157	LGMDR15, LGMD2O	
		Retinitis pigmentosa 76—617,123		
*5*	*POMGNT2* (protein O-linked-mannose β−1,4-N-acetylglucosaminyltransferase 2)—614,828	MDDGA8—614,830		*GTDC2* (glycosyltransferase-like domain-containing protein 2), *C3ORF39* (chromosome 3 open reading frame 39)
		MDDGC8—618,135	LGMDR24	
*6*	*POMK* (protein O-mannose kinase)—615,247	MDDGA12—615,249		*SGK196* (sugen kinase 196)
		MDDGC12—616,094		
*7*	*FKTN* (Fukutin)—607,440	MDDGA4—253,800	FCMD	
		MDDGB4—613,152		
		MDDGC4—611,588	LGMDR13, LGMD2M	
		Cardiomyopathy, dilated, 1X—611,615		
*8*	*FKRP* (fukutin-related protein)—606,596	MDDGA5—613,153		
		MDDGB5—606,612	MDC1C	
		MDDGC5—607,155	LGMDR9, LGMD2I	
*9*	*LARGE 1* (acetylglucosaminyltransferase-like protein 1)*—603,590*	MDDGA6—613,154		*LARGE*
		MDDGB6—608,840	CMD1D	
*10*	*RXYLT1 (*ribitol xylosyltransferase 1)—605,862	MDDGA10—615,041		*TMEM5* (transmembrane protein 5)
*11*	*B3GALNT2* (beta-1,3-N-acetylgalactosaminyltransferase 2)—610,194	MDDGA11—615,181		
*12*	*B4GAT1* (beta-1,4-glucuronyltransferase 1)—605,517	MDDGA13—615,287		*B3GNT1* (beta-1,3-N-acetylglucosaminyltransferase 1)
Tertiary DGPs				
*13*	*CRPPA (*CDP-L-ribitol pyrophosphorylase A)—614,631	MDDGA7—614,643		*ISPD* (isoprenoid synthase domain-containing protein)
		MDDGC7—616,052	LGMDR20, LGMD2U	
*14*	*GMPPB (*GDP-mannose pyrophosphorylase, beta subunit)—615,320	MDDGA14—615,350		
		MDDGB14—615,351		
		MDDGC14—615,352	LGMDR19, LGMD2T	
*15*	*DPM1 (*dolichyl-phosphate mannosyltransferase 1)—603,503	CDG, type Ie—608,799		*MPDS* (MDP synthase)
*16*	*DPM2 (*dolichyl-phosphate mannosyltransferase 2)—603,564	CDG, type Iu—615,042		
*17*	*DPM3 (*dolichyl-phosphate mannosyltransferase 3)—612,937	? MDDGB15*—618,992		
		MDDGC15—612,937		
*18*	*DOLK* (dolichol kinase)—610,746	CDG, type Im—610,768		*TMEM15* (transmembrane protein 15), *DK1* (dolichol kinase)

*CDG*, congenital disorder of glycosylation; *FCMD*, Fukuyama congenital muscular dystrophy; *LGMD*, limb-girdle muscular dystrophy; *MDDG*, muscular dystrophy-dystroglycanopathy

^*^Compound heterozygous variants have been identified in a Chinese girl the DPM3 gene by a targeted gene panel. Variants were segregated in the family, but no functional studies of the variants were performed(Carss et al. 2013)

The most severe form of the clinical spectrum of DGPs is known as congenital muscular dystrophy-dystroglycanopathy with brain and eye anomalies type A (MDDGA), previously designated Walker-Warburg syndrome (WWS) or muscle-eye-brain disease (MEB). The intermediate phenotype of the spectrum is represented by congenital muscular dystrophy-dystroglycanopathy with or without mental retardation type B (MDDGB). The mildest end of the phenotypic spectrum is defined as limb-girdle muscular dystrophy-dystroglycanopathy type C (MDDGC).

Given the clinical and genetic heterogeneity of DPGs, short-read exome sequencing (WES) represents a rapid, cost-effective, and accurate method for their diagnosis with variant detection in about 50% of cases Johnson et al. 2018. Short-read sequencing is limited by the short length of the generated reads (50 to 300 base pairs), where some reads cannot be mapped to the reference genome. Such reads may be discarded and create gaps in the sequencing data accounting for the relatively low diagnostic rate. Consistently, it has been evident that long-read sequencing which can read longer lengths of 5000 to 30,000 base pairs can potentially enhance the diagnostic rates in neuromuscular disorders Owusu and Savarese 2023. Transcriptome sequencing via RNA-Seq can also boost neuromuscular diagnoses and prioritize reported variants (Marchant et al. 2024, Segarra-Casas et al. 2024). Whether such advanced techniques would contribute to elevated diagnostic rates in patients with DGPs should be addressed in the upcoming studies.

Accurate diagnosis is crucial to explain the patient’s health problem and to guide the subsequent healthcare decisions. Disclosing the genetic cause can also contribute to limiting disease incidence via premarriage, prenatal, and preimplantation genetic testing. It may also pave the way to identify new clinical findings, elaborate distinct genotype–phenotype correlations, and characterize novel genes, particularly in large inbred ones Johnson et al. 2018, 

Cubilla et al. 2023. On the other hand, several therapeutic interventions for DGPs, including gene and antisense therapies, have been proposed with promising consequences in the preclinical studies Taniguchi-Ikeda et al. 2016, Zambon et al. 2024, where identifying the genetic cause is a cornerstone for enrolment into clinical trials.

### Congenital Muscular Dystrophy-Dystroglycanopathy with Brain and Eye Anomalies Type A (MDDGA)

The phenotype is characterized by progressive muscular dystrophy and profound mental retardation with relatively severe brain and eye malformations. Defective features of brain structures involve lissencephaly, hydrocephalus, agenesis of the corpus callosum, cerebellar hypoplasia or dysplasia, cerebellar cysts, ventricular dilatation, vermis hypoplasia, and flattening of the pons and brainstem. Ocular abnormalities include microphthalmia, buphthalmos, cataract, myopia, corneal opacity, retinal dysplasia, and glaucoma. Other recurrent features are neural tube defects, seizures, hypotonia, cleft lip/palate, and sensorineural hearing loss (Godfrey et al. 2007, Clement et al. 2008, Roscioli et al. 2012). Both micro- and macrocephaly can be reported with a higher incidence of microcephaly Costanzo et al. 2014, Al Dhaibani et al. 2018.

MDDGA includes both Walker-Warburg syndrome (WWS) and muscle-eye-brain disease (MEB), where brain abnormalities are less severe in MEB than in WWS Godfrey et al. 2007. Patients with WWS often die within the first year of life, whereas those with MEB disease may scarcely gain the ability to walk and speak a few words Dobyns et al. 1989. To date, MDDGA has been reported in association with variants in 14 genes: *POMT1* (MDDGA1; 236670), *POMT2* (MDDGA2; 613150), *POMGNT1* (MDDGA3; 253280), *FKTN* (MDDGA4; 253,800), *FKRP* (MDDGA5; 613153), *LARGE1* (MDDGA6; 613154), *CRPPA* (MDDGA7; 614643), *POMGNT2* (MDDGA8; 614830), *DAG1* (MDDGA9; 616538), *RXYLT1* (MDDGA10; 615,041), *B3GALNT2* (MDDGA11; 615181), *POMK* (MDDGA12; 615249), *B4GAT1* (MDDGA13; 615287), and *GMPPB* (MDDGA14; 615350).

### Congenital Muscular Dystrophy-Dystroglycanopathy With or Without Mental Retardation Type B (MDDGB)

It is characterized by congenital muscular dystrophy with moderate or mild structural brain abnormalities. Impaired cognition is evident in almost all patients Mercuri et al. 2009, Godfrey et al. 2007. In the majority of cases, eye anomalies are either absent or relatively mild including myopia, strabismus, and cataract. Common disease features are hypotonia, joint contractures, microcephaly, epilepsy, and cardiac dysfunction Clement et al. 2008, Reeuwijk et al. 2006, Clarke et al. 2011, Carss et al. 2013. To date, the MDDGB phenotype has been attributed for variants in only eight genes; *POMT1* (MDDGB1; 613155), *POMT2* (MDDGB2; 613156), *POMGNT1* (MDDGB3; 613151), *FKTN* (MDDGB4; 613152), *FKRP* (MDDGB5; 616612), *LARGE1* (MDDGB6; 608840), *GMPPB* (MDDGB14; 615351), and *DPM3* (MDDGB15; 618992).

### Limb-Girdle Muscular Dystrophy-Dystroglycanopathy Type C (MDDGC)

In the limb-girdle phenotype, patients acquired early motor milestones, precluding congenital muscular dystrophy. The age at onset varies widely from early infancy to adulthood with muscle weakness and motor difficulties, where ambulation may be lost with advancing age Tasca et al. 2013, Mercuri et al. 2003. Noteworthy, pathogenic variants were reported in clinically asymptomatic subjects with calf hypertrophy and high CK levels Endo et al. 2015. Patients may exhibit variable degrees of cognitive delay; however, normal intellectual development was also reported in a significant proportion of cases. Brain and eye anomalies are uncommon among patients with MDDGC. The MDDGC phenotype has been currently correlated with pathogenic variants in 11 different genes: *POMT1* (MDDGC1; 609308), *POMT2* (MDDGC2; 613158), *POMGNT1* (MDDGC3; 613157), *FKTN* (MDDGC4; 611588), *FKRP* (MDDGC5; 607155), *CRPPA* (MDDGC7; 616052), *POMGNT2* (MDDGC8; 618135), *DAG1* (MDDGC9; 613818), *POMK* (MDDGC12; 616094), *GMPPB* (MDDGC14; 615352), and *DPM3* (MDDGC15; 612937).

Further delineation of more patients would extend the clinical spectrum associated with each gene. It could be suggested that a distinct phenotype is not restricted to a specific array of genes. According to the Online Mendelian Inheritance in Man (OMIM) database, diseases associated with *DOLK*, *DPM1*, and *DPM2* variants are designated as congenital disorders of glycosylation, type Im (CDG1M; 610768), type Ie (CDG1E; 608799), and type Iu (CDG1U) (615042), respectively. However, patients reported with pathogenic variants in these genes exhibited phenotypes similar to MEB disease Radenkovic et al. 2021. Noteworthy, variants in *DPM3* have often been identified in patients with isolated muscle dystrophy and cardiomyopathy Nagy et al. 2022 and to a lesser extent in patients with combined muscle and central nervous system (CNS) involvement Fu et al. 2019.

### Other Diseases Associated with Dystroglycanopathies-Related Genes

Notably, pathogenic variants in genes correlated with DGPs can cause distinct phenotypes rather than muscular dystrophies. In those circumstances, the genetic variant was mainly revealed by NGS highlighting the unique avail of its application in unraveling the molecular defects in genetic diseases. Extending the phenotypic spectrum associated with genes responsible for α-DG glycosylation might illuminate their intricate mechanistic pathways in different tissues and developmental stages. Of course, environmental and epigenetic factors might impact the patient’s phenotype.

*POMGNT1* variants have been associated with the different forms of DGPs, and they have also been specified as a cause of retinitis pigmentosa in some patients Patel et al. 2023, Xu et al. 2016. Interestingly, a deficiency of *B3GALNT2* was reported in a girl with neurodevelopmental delay without any symptoms of muscle weakness D’haenens et al., 2022*FKTN* defects resulted in dilated cardiomyopathy in four unrelated families, where affected patients had no or mild limb-girdle muscle involvement with normal cognition Murakami et al. 2006.

### Therapeutic Approaches

Despite advances in the molecular pathophysiology of DGPs, there is still no therapeutic approach that can provide an effective cure for this group of disorders. As the most common form of DGPs Nallamilli et al. 2018, *FKRP*-associated disease is notably addressed in most studies with several developed animal and human models Ortiz-Cordero et al. 2021a.

### Pharmacological Agents

Several pharmacological agents have shown beneficial effects through general mechanisms irrespective of the mutant gene. Corticosteroids provide the gold standard of care in Duchenne Muscular Dystrophy (DMD). They control patients’ symptoms and delay disease progression by virtue of their strong anti-inflammatory action Kourakis et al. 2021. Consistently, they improved muscle pathology in *FKRP*-mutant mice Wu et al. 2016 as well as in *GMPPB* mutant patient Fecarotta et al. 2018. Bisphosphonates are widely applied to increase bone density in osteoporosis with limited effect on muscles. However, they significantly enhanced the therapeutic effect of corticosteroids in the *FKRP* mouse model increasing muscle function and strength Wu et al. 2016. Selective estrogen receptor modulators (SERMs) are a class of drugs that block estrogen receptors (ERs) with anti-inflammatory and anti-fibrotic effects. Their long-term administration markedly improved muscle strength, as well as cardiac and respiratory functions in *FKRP*-deficient mice Wu et al. 2018. Rapamycin is a potent immunosuppressant drug that inhibits the mammalian target of rapamycin (mTOR). mTOR is a highly conserved serine/threonine protein kinase that acts as a critical regulator of skeletal muscle mass through controlling protein metabolism Bodine 2022. The mTOR signaling was found to be activated upon muscle dystrophy Eghtesad et al. 2011, Ramos et al. 2012. Rapamycin administration reduced fibrosis, inflammation, and muscle damage, while increasing the size of muscle fibers in *FKTN* knockout mice Foltz et al. 2016. Pentetic acid binds and inactivates metallic ions, such as calcium and magnesium. It decreased muscle and cardiac pathologies in *FKRP*-mutant zebrafish Serafini et al. 2018. Supplementation of the coenzyme nicotinamide adenine dinucleotide (NAD +) prior to muscle development enhanced muscle structure and function in *FKRP* zebrafish morphants Bailey et al. 2019. 4BPPNit is a small molecule that potentially augmented the glycosylation of α-DG, partially due to upregulation of *LARGE1* expression Kim et al. 2019. A cluster of other molecules also positively modulated α-DG glycosylation in patient-derived myoblasts Lv et al. 2015.

Specific treatment strategies depending on the defective gene have been developed for distinct types of DGPs. Fukuyama congenital muscular dystrophy (FCMD) is a distinct type of DGPs that mainly exists in Japan Kobayashi et al. 1998. It occurs due to the ancestral insertion of a 3-kb transposon in the 3′-untranslated region of the *FKTN* gene, resulting in abnormal gene splicing Taniguchi-Ikeda et al. 2011a. Administration of antisense nucleotides able to correct this splicing abnormality restores the normal function of *FKTN* in both mice model and patient-derived cells Enkhjargal et al. 2023, Taniguchi-Ikeda et al. 2011b. CDP-Rbo, synthesized by CRPPA, serves as a donor substrate of RboP for FKTN and FKRP. Therefore, *CRPPA* defects inhibit the FKTN- and FKRP-dependent transfer of the RboP onto core M3. Supplementation of Rbo and its precursor ribose (Rib) enhanced the enzymatic activity of both *CRPPA* Tol et al. 2019, Kanagawa et al. 2016, Tokuoka et al. 2022 and *FKRP* Cataldi et al. 2018, Ortiz-Cordero et al. 2021, Thewissen et al. 2024 in deficient models, where an increased level of enzyme substrate promotes the activity of the mutant enzyme. Notably, this effect is directly proportional to the residual enzyme activity. The therapeutic effect of Rbo and Rib was enhanced by NAD + in human *FKRP*-mutant myotubes Ortiz-Cordero et al. 2021. Rbo therapy is expected to have similar effects in *FKTN* deficient models provided that residual enzyme activity is present. Phase 2 (NCT04800874) and phase 3 (NCT05775848) clinical trials evaluating the safety and efficacy of ribitol (BBP-418) administration in patients with *FKTN* variants are ongoing with preliminary positive results Harper et al. 2022.

### Gene and Cell-Based Therapy

As single gene disorders, gene therapy would provide an effective therapeutic approach for DGPs. Delivery of a functional gene copy using viral vectors (AAV) was found to ameliorate disease pathology in *FKRP* Xu et al. 2013, Vannoy et al. 2018, Vannoy et al. 2019, Qiao et al. 2014, Gicquel et al. 2017, Frattini et al. 2017, Cataldi et al. 2023 as well as *FKTN* Kanagawa et al. 2013 and *LARGE1* Barresi et al. 2004, Yu et al. 2013, Yonekawa et al. 2022 deficient mice. Interestingly, in utero electroporation of *FKTN* and *LARGE1* into the brains of fetal mice inhibited severe brain deformity in *FKTN* and *LARGE1* mutant mice, respectively Sudo et al. 2018. Two phase 1–2 clinical trials, NCT05230459 and NCT05224505, have been launched to estimate the tolerability and efficiency of intravenous injection of adeno-associated viral vector (AAV) carrying functional *FKRP* gene in patients with *FKRP* variants.

Some studies demonstrated that overexpression of distinct genes can compensate for the loss of other genes involved in α-DG glycosylation. For example, *LARGE1* overexpression was able to rescue the phenotypes associated with *POMT1*, *POMGNT1*, *FKTN*, and *FKRP* variants providing a universal approach for DGP treatment Barresi et al. 2004, Yu et al. 2013, Kanagawa et al. 2009, Vannoy et al. 2014. However, systemic upregulation of *LARGE1* is known to aggravate muscular dystrophy in mice models Saito et al. 2014, Whitmore et al. 2013. Overexpression of *CRPPA* and *B4GALNT2* also revealed a significant reduction of muscle pathology in *FKRP*-mutant mice Thomas et al. 2016, Cataldi et al. 2020.

Gene correction of patient-specific induced pluripotent stem (iPS) cells using the CRISPR-Cas9 technology has recently emerged as a promising approach for autologous cell therapy in DGPs Kim et al. 2019, Dhoke et al. 2021. Intramuscular injection of murine and human myogenic progenitors into the *FKRP*-mutant mouse model also improved the muscle phenotype of dystrophic *FKRP*-mutant mice Azzag et al. 2020.

## Concluding Remarks

DGPs arise due to defective glycosylation of α-DG with functional and/or structural involvement of CNS and eyes. α-DG is heavily glycosylated forming three distinct core motifs with various structures. However, inserting GlcA-Xyl repeating disaccharide units, known as matriglycan, at the final phase of core M3 maturation, is the only prerequisite for α-DG binding with LG domain-containing proteins. Consistently, DGPs have been found to originate due to defects in genes necessary for the generation of core M3. The only exception is *POMGNT1* which is responsible for synthesis of core M1 (not M3); however, its deficiency has also been documented in many patients with DGPs. This would be explained as it guides FTKN to the core M3 enabling the glycan processing. A low diagnostic rate (about 50%) represents a critical issue of DGPs. Robust advances in molecular techniques would enable variant detection in already known genes and/or discover novel genes or disease mechanisms in this distinct group of neuromuscular diseases. In turn, this would contribute to the desired hope of finding an effective therapy.

## Data Availability

No datasets were generated or analysed during the current study.
